# Complete Genome Sequences of 17 Canadian Isolates of *Salmonella enterica* subsp. *enterica* Serovar Heidelberg from Human, Animal, and Food Sources

**DOI:** 10.1128/genomeA.00990-16

**Published:** 2016-09-15

**Authors:** Geneviève Labbé, Kim Ziebell, Sadjia Bekal, Kimberley A. Macdonald, E. Jane Parmley, Agnes Agunos, Andrea Desruisseau, Danielle Daignault, Durda Slavic, Linda Hoang, Danielle Ramsay, Frank Pollari, James Robertson, John H. E. Nash, Roger P. Johnson

**Affiliations:** a National Microbiology Laboratory at Guelph, Public Health Agency of Canada, Guelph, Ontario, Canada; b Laboratoire de Santé Publique du Québec, Sainte-Anne-de-Bellevue, Québec, Canada; c National Microbiology Laboratory at Winnipeg, Public Health Agency of Canada, Winnipeg, Manitoba, Canada; d British Columbia Centre for Disease Control, Public Health Microbiology and Reference Laboratory, Vancouver, British Columbia, Canada; e Centre for Foodborne, Environmental and Zoonotic Infectious Diseases, Public Health Agency of Canada, Guelph, Ontario, Canada; f National Microbiology Laboratory at St-Hyacinthe, Public Health Agency of Canada, St-Hyacinthe, Québec, Canada; g Animal Health Laboratory, Laboratory Services Division, University of Guelph, Guelph, Ontario, Canada; h Ministère de l’Agriculture, des Pêcheries, et de l’Alimentation du Québec, Québec, Canada; i National Microbiology Laboratory at Toronto, Public Health Agency of Canada, Toronto, Ontario, Canada

## Abstract

*Salmonella enterica* subsp. *enterica* serovar Heidelberg is a highly clonal serovar frequently associated with foodborne illness. To facilitate subtyping efforts, we report fully assembled genome sequences of 17 Canadian *S*. Heidelberg isolates including six pairs of epidemiologically related strains. The plasmid sequences of eight isolates contain several drug resistance genes.

## GENOME ANNOUNCEMENT

We present closed genome sequences of 17 Canadian isolates of *Salmonella enterica* subsp. *enterica* serovar Heidelberg collected between 2003 and 2014 in Ontario, Quebec, and British Columbia. Twelve isolates comprise six epidemiologically related pairs (Table 1): three pairs from outbreaks in Quebec in 2012, 2013, and 2014 (1); one pair from an outbreak in British Columbia in 2003; and two pairs collected from broiler chicken feces in Ontario in 2013 as part of the Canadian Integrated Program for Antimicrobial Resistance Surveillance. The five remaining isolates are unrelated and include two isolates from retail chicken meat, one from chicken cecal contents collected at a slaughterhouse, as well as two animal clinical isolates.

**TABLE 1:** Accession numbers and metadata for the genomes of 17 *Salmonella* serovar Heidelberg isolates sequenced in this study

GenBank accession no.	Local reference ID	Pair ID, source^a^	AMR profile (resistance genes in plasmids according to ResFinder 2.0)	PFGE profile (XbaI/BlnI)	Phage type
CP016565	AMR588-04-00318	A, chicken feces	AcAmCeCfCxSt *(blaCMY-2)*	SHEXAI.0001/SHEBNI.0001	29
CP016569	AMR588-04-00320	A, chicken feces	AcAmCeCfCxSt *(blaCMY-2)*	SHEXAI.0001/SHEBNI.0001	29
CP016573	AMR588-04-00435	B, chicken feces	Susceptible	SHEXAI.0001/SHEBNI.0001	19
CP016576	AMR588-04-00437	B, chicken feces	Susceptible	SHEXAI.0001/SHEBNI.0001	19
CP016507	SH12-003	C, human	Susceptible	SHEXAI.0001/SHEBNI.0001	19
CP016504	SH12-007	C, human	Susceptible	SHEXAI.0001/SHEBNI.0001	19
CP016579	SH13-006	D, human	Susceptible	SHEXAI.0001/SHEBNI.0001	26
CP016586	SH13-004	D, human	Susceptible	SHEXAI.0001/SHEBNI.0001	26
CP016510	SH14-028	E, food	Susceptible	SHEXAI.0001/SHEBNI.0001	19
CP016581	SH14-009	E, human	GeSt [*aac(3)-VIa, aadA1*]	SHEXAI.0001/SHEBNI.0001	19
CP016561	A3ES40	F, food	Susceptible	SHEXAI.0001/SHEBNI.0001	26
CP016563	A3EZ223	F, human	Susceptible	SHEXAI.0001/SHEBNI.0001	26
CP016525	09-036813-1A	NA, equine clinical	AmChGeKaSuTm [*strB, strA, aac(6′)-IIc, aph(3′)-Ia, blaTEM-1B, QnrB49, ere(A), sul1, dfrA18*]	SHEXAI.0014/SHEBNI.0210	29
CP016514	11-004736-1-7	NA, bovine clinical	AcAmCeCfCx *(blaCMY-2)*	SHEXAI.0001/SHEBNI.0001	29
CP016530	SA01AB09084001	NA, chicken cecal contents	AcAmCeCfCx *(blaCMY-2)*	SHEXAI.0001/SHEBNI.0001	19
CP016521	SA02DT09004001	NA, chicken meat	AcAmCeCfCx *(blaCMY-2)*	SHEXAI.0001/SHEBNI.0001	9
CP016517	CE-R2-11-0435	NA, chicken meat	Am *(blaTEM-1B)*	SHEXAI.0001/SHEBNI.0001	20

a Six pairs (A to F) of related strains are shown in paired rows and are from broiler chicken feces collected in 2013 (pairs A and B) and from foodborne illness outbreaks that occurred in 2012 (pair C), 2013 (pair D), 2014 (pair E), and 2003 (pair F). NA, not applicable.

Genomic DNA was extracted using either the EZ1 DNA tissue kit or the DNeasy 96 blood and tissue kit (Qiagen, Hilden, Germany). Sequencing was performed on two platforms: (1) PacBio (using SMRT cells in RSII sequencers [2]), which generated 79,000 to 190,000 raw subreads averaging 4,700 to 11,600 bp with 92 to 222× coverage that were assembled into contigs by the sequencing facilities using the HGAP workflow; (2) and/or Illumina after library preparation with the Illumina Nextera XT DNA library preparation kit, using either Illumina MiSeq with 2 × 251 paired-end runs achieving 47 to 131× coverage, and/or Illumina HiSeq with 2 × 101 paired-end runs achieving 390 to 615× coverage. The Illumina reads were analyzed and quality-checked using FastQC (http://www.bioinformatics.babraham.ac.uk/projects/fastqc/). Genomes were assembled with MIRA assembler version 4.9.3 (3), with the Illumina reads assembled to the PacBio consensus sequence for each isolate to correct errors, and by manually checking potential joins using the Gap5 Software v1.2.14 of the Staden package (4). In contrast with the other strains, the isolates from outbreak C (Table 1) were sequenced with both Illumina HiSeq and Illumina MiSeq and not with PacBio; in this case, the closed genome sequence from strain CFSAN002064 (GenBank accession no. NZ_CP005995) (5) was used for the initial reference assembly of the Illumina reads. Plasmids were assembled as previously described (6). Comparison of the genome assemblies with the genome optical maps of other *S*. Heidelberg strains found in GenBank (6), together with the finishing process produced fully assembled genomes. The genomes consisted of single chromosomal contigs ranging from 4,747,525 to 4,751,746 bp, with an average G+C content of 52.19%. The genomes were annotated with the National Center for Biotechnology Information (NCBI) Prokaryotic Genomes Annotation Pipeline (PGAP) (http://ncbi.nlm.nih.gov/genomes/static/Pipeline.html), identifying an average of ~4,596 coding DNA sequences (CDS) per genome.

The pulsed-field gel electrophoresis (PFGE) profiles, phage types and antimicrobial resistance profiles of all isolates were determined by established methods (7). Eight of the 17 isolates were drug resistant (Table 1) with plasmid-borne resistance genes. The two strains of pair E (Table 1) had different AMR plasmid content, as has been reported previously for *S*. Heidelberg (8).

### Accession number(s).

The complete genome sequences of these 17 isolates of *S*. Heidelberg as well as the complete sequences of their 40 plasmids have been deposited in GenBank under BioProject no. 298211 and 305824. The GenBank accession numbers for the genomes are listed in Table 1.

## References

[1] Bekal S, Berry C, Reimer AR, Van Domselaar G, Beaudry G, Fournier E, Doualla-Bell F, Levac E, Gaulin C, Ramsay D, Huot C, Walker M, Sieffert C, Tremblay C 2016 Usefulness of high-quality core genome single-nucleotide variant analysis for subtyping the highly clonal and the most prevalent *Salmonella enterica* serovar Heidelberg clone in the context of outbreak investigations. J Clin Microbiol 54:289–295. doi:10.1128/JCM.02200-15. 26582830 PMC4733192

[2] Chin CS, Alexander DH, Marks P, Klammer AA, Drake J, Heiner C, Clum A, Copeland A, Huddleston J, Eichler EE, Turner SW, Korlach J 2013 Nonhybrid, finished microbial genome assemblies from long-read SMRT sequencing data. Nat Methods 10:563–569. doi:10.1038/nmeth.2474. 23644548

[3] Chevreux B, Wetter T, Suhai S 1999 Genome sequence assembly using Trace signals and additional sequence information, p. 45–56. *In* Proceedings of the German Conference on Bioinformatics, vol. 99 Computer Science and Biology, Hannover, Germany.

[4] Staden R, Beal KF, Bonfield JK 2000 The Staden package, 1998. Methods Mol Biol 132:115–130. 10547834 10.1385/1-59259-192-2:115

[5] Evans PS, Luo Y, Muruvanda T, Ayers S, Hiatt B, Hoffman M, Zhao S, Allard MW, Brown EW 2014 Complete genome sequences of *Salmonella enterica* serovar Heidelberg strains associated with a multistate food-borne illness investigation. Genome Announc 2(3):e01154-13. doi:10.1128/genomeA.01154-13. 24903882 PMC4047461

[6] Labbé G, Edirmanasinghe R, Ziebell K, Nash JH, Bekal S, Parmley EJ, Mulvey MR, Johnson RP 2016 Complete genome and plasmid sequences of three Canadian isolates of *Salmonella enterica* subsp. *enterica* serovar Heidelberg from human and food sources. Genome Announc 4(1):e01526-15. doi:10.1128/genomeA.01526-15. 26769926 PMC4714108

[7] Jokinen CC, Koot J, Cole L, Desruisseau A, Edge TA, Khan IU, Koning W, Lapen DR, Pintar KD, Reid-Smith R, Thomas JL, Topp E, Wang LY, Wilkes G, Ziebell K, van Bochove E, Gannon VP 2015 The distribution of *Salmonella enterica* serovars and subtypes in surface water from five agricultural regions across Canada. Water Res 76:120–131. doi:10.1016/j.watres.2015.02.038. 25799976

[8] Hoffmann M, Zhao S, Pettengill J, Luo Y, Monday SR, Abbott J, Ayers SL, Cinar HN, Muruvanda T, Li C, Allard MW, Whichard J, Meng J, Brown EW, McDermott PF 2014 Comparative genomic analysis and virulence differences in closely related *Salmonella enterica* serotype Heidelberg isolates from humans, retail meats, and animals. Genome Biol Evol 6:1046–1068. doi:10.1093/gbe/evu079. 24732280 PMC4040988

